# Intestinal Obstruction—Gastrotomy—Death

**Published:** 1874-01

**Authors:** 


Intestinal Obstruction—Gastrotomy—Death.—D. W. Cheever, M.D., reports: I. G., a boy of seven years, suddenly attacked; pain in abdomen, obstinate constipation, vomiting, tympanites and tenderness. Purgatives and enemata used without effect. Repeated punctures with aspirator to relieve the great distension. Eighth day, abdomen was opened. There was moderate peritonitis. No effusion in the abdomen. The puncture marks in the intestines were seen, closed up, without extravasion or external redness. The obstruction was found in the illium, just above the cæcum. The illium was much distended above the point, but empty and collapsed below, and so was the large intestine. There was no invagination or intussusception. The illium was twisted and held by a band of adhesion which looked old. This was easily severed, and the bowel untwisted. Air immediately passed through and then followed, at once, an enormous liquid and gaseous discharge *per anum.* The abdomen was now closed as rapidly as possible, warmth applied and stimulants given. The child did not rally, but died, in a state of collapse, after three hours.

